# Editorial: Crossmodal correspondence

**DOI:** 10.3389/fpsyg.2024.1385480

**Published:** 2024-03-21

**Authors:** Na Chen, Thomas Alrik Sørensen, Charles Spence

**Affiliations:** ^1^The Gonda Multidisciplinary Brain Research Center, Bar-Ilan University, Ramat Gan, Israel; ^2^Department of Communication and Psychology, Aalborg University, Aalborg, Denmark; ^3^Department of Experimental Psychology, University of Oxford, Oxford, United Kingdom

**Keywords:** crossmodal correspondences, sound-shape, music-taste, development, color-taste

Over the last decade or so, there has been an explosion of scientific research interest in the crossmodal correspondences, this the name given to the often surprising, yet consensual, associations that have been documented between a growing number of basic sensory features, attributes, and dimensions in different sensory modalities (see Spence, 2011, for a review). So, for example, people have been shown to match auditory pitch with visual elevation, size, lightness, etc. Over the years, several different mechanisms have been put forward to help explain the existence of such crossmodal correspondences, including the statistical account, the structural (or neurophysiological) account, a semantic/lexical account, and an account in terms of emotional-mediation. It is, however, important to note that these various explanations should not be treated as mutually exclusive. Indeed, several or perhaps all of them may help to explain a variable proportion of the various different correspondences that have been documented to date.

The 13 papers that were eventually accepted for this Research Topic effectively serve to highlight the global growth of research interest in this emerging phenomenon currently. What is also striking is how the articles collected together here range well-beyond the pitch-based audiovisual correspondences that attracted so much of the interest in correspondences research previously (see Spence and Sathian, 2020, for a review). Recently, researchers have increasingly started to investigate group differences in crossmodal correspondences as well as studying when in human development humans start to express a sensitivity to such crossmodal correspondences (see Spence, 2022, for a review). The latter approach is illustrated in this Research Topic by an exploratory study reported by Meng et al. in which crossmodal correspondences between visual features (such as shape/angularity and color) and tastes (e.g., bitter, sweet, sour, salty) were assessed in a group of pre-schoolers. Importantly, several important factors that have been suggested to affect/constrain the crossmodal correspondences include their context-dependence (Motoki and Velasco, 2021), their automaticity (Spence and Deroy, 2013b; Getz and Kubovy, 2018), and their bidirectionality (Motoki et al., 2023; Yang et al., 2023; Chen and Huang). These topics are addressed in several of the contributions here. Chen and Huang showed that the sound-shape correspondences are not completely automatic, but their modulation was bidirectionally symmetrical once it occurred. The bidirectionality of crossmodal correspondences means that effects on visual perception can be elicited by stimuli from other sensory modalities, such as gustatory and/or olfactory stimuli, as highlighted by a couple of the intriguing submissions in this Research Topic (e.g., Ward et al.; Yang et al., 2023). Ward et al. used an achromatic adjustment task to show that the presence of odors modulates color perception. Such crossmodal effects on vision are surprising inasmuch as vision is so often found to be the dominant sense in multisensory research (Hutmacher, 2019).

“Sonic seasoning”, the generic name for the modification of the taste and flavor based on listening to music that corresponds crossmodally to the dominant tastes/flavors of food and drink that have been documented between sound and the chemical senses also continues to attract much research interest. The experimental paper from Xu et al. investigates the role of self-construal priming on the effectiveness of sonic seasoning. Meanwhile, the paper from Mesz et al. investigates emotional associations with music-based crossmodal correspondences.

Historically, the role of crossmodal correspondences in helping to solve the crossmodal binding problem has occupied the attention of many researchers (Chen and Spence, 2017). At the same time, the paper by Yang et al. (2023) demonstrates that the crossmodal correspondence between color and taste influences performance on the well-established Stroop task. However, what is striking about so many of the articles that are collected together here is how they attempt to apply our growing understanding of the crossmodal correspondences to a range of real-world applications: this includes everything from a consideration of the potential facilitatory role of designing human augmentation systems based on the crossmodal correspondences (see Pinardi et al.), through to the use of crossmodal correspondences to help describe, communicate about, and market wine (Crichton-Fock, Spence, Mora, et al.; Crichton-Fock, Spence, and Pettersson). At the same time, the crossmodal correspondences clearly also help to provide guidelines for the design of multisensory experiential events (see Velasco and Spence, 2022, for a review). Meanwhile, the article from Ogata et al. reports some intriguing results concerning the impact of the shape of chocolate on taste ratings, based on the literature on shape-taste crossmodal correspondences (see Spence, 2014).

One other area of continuing research interest concerns the relationship between crossmodal correspondences, synaesthesia, and mental imagery (Rader and Tellegen, 1987; Martino and Marks, 2001; Spence and Deroy, 2013a; Nanay, 2020). Relevant here, the paper by Hitsuwari and Nomura details research validating a Japanese version of the Plymouth Sensory Imagery Questionnaire which provides researchers with a means of assessing the strength of mental imagery in each of the senses.

Extending the scope of crossmodal correspondences research, this Research Topic also includes a couple of papers that might best be classified as sensory-conceptual/categorical correspondence (Chen et al.) and intramodal visual correspondences (Zelazny et al.). The first of these two papers demonstrates that the color red biases sex categorization of human bodies. One theme that emerges from the latter study, as well as from several other studies that have been published recently (e.g., Velasco et al., 2023) highlights the importance of providing participants with a wide enough range of options if one's goal is to identify the strongest correspondences, given that older studies with a narrow range of colors, say, may merely have picked up the best color amongst the range of options provided to the participant. Such methodological developments should help ensure that future theorizing about the correspondences is based on firm empirical foundations.

Taken together, the research papers that have been gathered together in this Research Topic clearly highlight the vibrant state of crossmodal correspondences research in both the theoretical and applied arenas. One exciting area in correspondences research that is not represented here relates to the emergence of studies assessing the sensitivity of various animals to crossmodal correspondences. So, for example, Loconsole et al. (2021, 2022) have recently published several studies demonstrating audiovisual crossmodal correspondences in both chicks and the tortoise (*Testudo hermanni*; Loconsole et al., 2023).

## Author contributions

NC: Writing – review & editing. TS: Writing – review & editing. CS: Writing – original draft, Writing – review & editing.
